# Cognitive reappraisal and affective response to physical activity: associations with physical activity behavior

**DOI:** 10.1186/s13104-024-06843-3

**Published:** 2024-07-02

**Authors:** Ceren Gürdere, Julina Sorgenfrei, Ines Pfeffer

**Affiliations:** 1 Department of Psychology, Bilkent University, Ankara, Turkey; 2 Department of Psychology, Medical School Hamburg, Hamburg, Germany; 3 Department of Pedagogy, Medical School Hamburg, Hamburg, Germany; 4 Institute of Cognitive and Affective Neuroscience (ICAN), Medical School Hamburg, Am Kaiserkai 1, 20457 Hamburg, Germany

**Keywords:** Cognitive reappraisal, Exercise, Affect, Valence, Emotion regulation

## Abstract

**Objective:**

Cognitive reappraisal (CR), as an adaptive emotion regulation strategy, may play a role in transforming affect in a positive direction during or after exercise, thereby supporting physical activity (PA) adherence. The present study aimed to test the associations among PA, CR frequency, and affective response to PA, and further to examine the role of CR on PA behavior through affective response.

**Methods:**

A cross-sectional study was conducted with a sample of 105 adults, 74 of whom were women, with a mean age of 25.91. Self-report scales were used to measure PA, CR, and affective response to PA. Along with scales, demographic questions on age, sex, and education level were included. Data was collected via an online questionnaire.

**Results:**

The frequency of CR use was positively associated with affective response, and affective response with PA behavior. Mediation analysis revealed that affective response mediated the relationship between CR and PA.

**Discussion:**

Results were in the expected direction demonstrating the mediating role of affective response between CR and PA which implies that PA adherence might be facilitated by CR engagement. PA intervention programs should consider implementing CR ability and use frequency improving techniques.

## Introduction

Physical inactivity has become a major public health problem in the past decades and ranks as the fourth leading risk factor for mortality worldwide [1, 2]. While the relationship between physical activity (PA) and physical and mental health is well established [3, 4], the underlying psychological mechanisms contributing to formation and maintenance of PA habits and the factors associated with physical inactivity still need to be better understood.

As individuals may tend to repeat activities that create pleasure and tend to avoid activities associated with displeasure or pain, affective response to exercise seems to be an important factor for exercise adherence [5–8]. Previous studies have demonstrated that judging past exercise experiences as pleasant or unpleasant influences future PA [5–10]. Moreover, positive affective states at the end of the exercise might be beneficial for the formation of exercise habits [8, 11, 12]. However, there is substantial evidence that individuals differ in their affective responses to PA [8, 13]. Previous research revealed that different exercise intensities lead to different affective changes, which varies across individuals [14, 15]. Thus, further explanations are required in order to understand why some individuals engage in moderate-to-vigorous exercise despite the negative affective changes that might arise during PA.

One factor explaining variation in affective responses to different exercise intensities could be the role of emotion regulation processes [16–18]. Cognitive reappraisal (CR) is one of the most effective emotion regulation strategies [18] and refers to being able to change one’s cognitions about a situation to reduce the emotional impact of that situation [19]. CR strategies are beneficial for changing one’s appraisal and perspective of an emotion-eliciting situation before fully experiencing it [19, 20], and have been found to be positively linked to subjective well-being [21, 22]. Although research interest in the role of emotion regulation in PA behavior has increased in recent years, to date, research on the relationship between PA and emotion regulation remains relatively scarce [16, 20]. For example, reappraisal of the initial negative affect during moderate-to-vigorous PA (MVPA) could be “It’s only for a few minutes and afterwards I will feel much better than before” or “The discomfort I feel actually shows that my muscles are getting stronger now”. CR is of particular interest because it is an adaptive emotion regulation strategy that can help transform affect during or after exercise into a positive (or less negative) affect [16, 23], which might support PA adherence.

### The present study

**Fig. 1:**
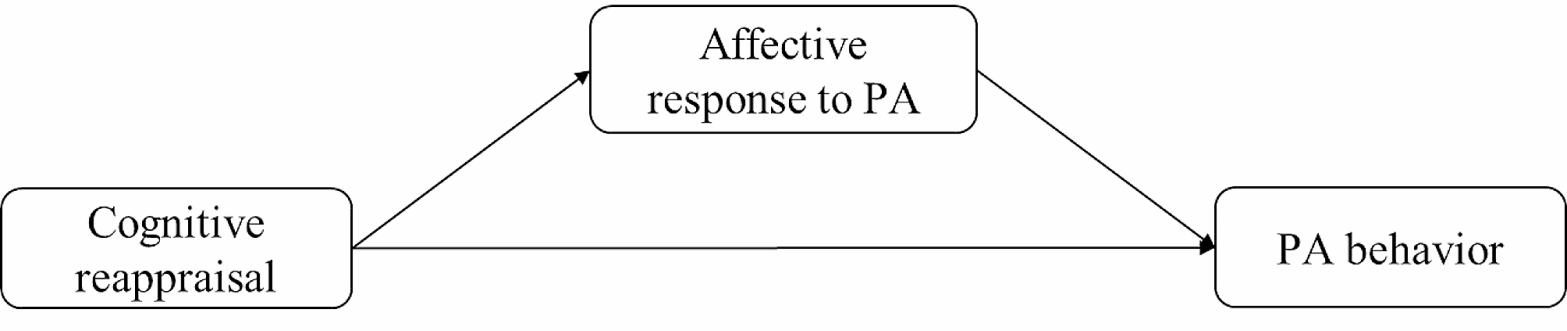
Expected associations between CR, affective response and PA behavior

The aim of the study was to examine the associations between CR and PA as well as the mediating role of AR (Fig. 1). We hypothesized that (1) CR is positively associated with PA behavior. Furthermore, we expected that (2) CR is positively associated with affective response to moderate as well as vigorous PA and (3) that affective response is positively correlated with PA behavior. Finally, it was expected that (4) affective response will act as a mediator between CR and PA.

## Methods

### Study design and procedure

A cross-sectional design with standardized self-report online questionnaires was employed (Unipark software; Tivian EFS Survey). After giving informed consent, participants filled in questions including socio-demographic information, PA behavior, affective response to PA, and the use of CR. Data collection ran between June and November 2022. Students received course credits in return for participation. To non-student participants no incentive was given.

### Participants

Participants (*N* = 105) were recruited via flyers posted on the university website, an online recruitment platform (SONA system), and social media channels. After the exclusion of outliers (+/- 3 SD), and insufficient or missing data, the final sample consisted of *N =* 101 participants (73.3% were women; *M*age = 25.91; SD = 6.95; range 18–58 years). 60 participants completed secondary education (56.5%), 41 university degree (39.6%), and 4 postgraduate degree (4.0%).

### Measures

CR was assessed using the CR subscale of the *Emotion Regulation Questionnaire* [24, German version by 25]. The subscale CR consists of 6 items (e.g. When I want to feel more positive emotion, I change the way I’m thinking about the situation) rated on a 7-point Likert scale ranging from 1 (*strongly disagree*) to 7 (*strongly agree*). An average score is calculated, where higher scores indicate greater frequency of using CR. The psychometric properties of the German version of the scale were acceptable; internal consistency (Cronbach’s alpha) of the questionnaire was reported as α = 0.74 [25].

Affective response (pleasure-displeasure continuum) to PA was measured using the one-item *Feeling Scale* [26, German version by 27]. After presenting the definition of moderate and vigorous PA, the participants were asked to rate their affect during moderate and vigorous PA separately, on a 11-point Likert scale ranging from − 5 (*very bad*) to + 5 (*very good*), with 0 indicating neutral affect. The convergent validity of the Feeling Scale German version ranges from *r* = .72 to 0.73 [27].

PA behavior as the dependent variable was measured using four items from the *International Physical Activity Questionnaire-Short Form* [28, German version by 29]. Participants were asked to indicate how many times and for how long (in minutes) on average per occasion they perform (1) moderate and (2) vigorous PA in a regular week. We calculated minutes per week spent with (1) moderate and (2) vigorous PA. A total weekly PA score (minutes/week) for MVPA was calculated by adding up the scores of moderate and vigorous PA. The questionnaire has acceptable measurement properties, with a good test-retest reliability of 0.80 and a fair to moderate criterion validity of rho = 0.30 [28].

### Data analysis

Statistical analyses were performed using the IBM SPSS Statistical Software (SPSS) Version 27 [30]. Descriptive statistics and bivariate correlations were computed. According to Cohen [31] effect sizes of correlations are considered to be small (*r* = .10), medium (*r* = .30), and large (*r* = .50). To test the mediation hypothesis, mediation analyses were conducted based on regression analyses and 10.000 bias corrected bootstraps using the PROCESS macro [32].

## Results

Descriptive statistics and bivariate correlations of all the study variables are presented in Table 1. In line with hypotheses 1–3, CR was significantly and positively but weakly associated with moderate PA and vigorous PA, and moderately with total MVPA. Furthermore, significant positive correlations were observed between CR and affective response to moderate (moderate effect) and vigorous PA (small effect). Affective response to moderate PA was significantly correlated with moderate and MVPA (both small to moderate effects) but not with vigorous PA, while affective response to vigorous PA was associated with vigorous (small effect) and MVPA (moderate effect) but not with moderate PA.

**Table 1:** Means, standard deviations and correlations among study variables (*N* = 101)

	1	2	3	4	5	6	7	8
1.Age	-							
2. Sex	− 0.11	-						
3. Cognitive Reappraisal	− 0.19	0.14	-					
4. AR to moderate PA	− 0.09	− 0.12	0.39***	-				
5. AR to vigorous PA	− 0.08	− 0.09	0.22*	0.51***	-			
6. Moderate PA (min/week)	0.05	0.09	0.23*	0.29**	0.20*	-		
7. Vigorous PA (min/week)	− 0.18	− 0.15	0.26**	0.13	0.34***	0.01	-	
8. MVPA (min/week)	− 0.11	− 0.06	0.34***	0.28*	0.39***	0.63***	0.79***	-
*M*	25.91	73.3^a^	4.80	8.72	7.77	108.33	148.33	256.65
*SD*	6.95	-	0.99	1.76	2.45	111.66	141.32	181.35

*Notes*. ^a^ % women; AR = affective response; PA = physical activity; MVPA = moderate-to-vigorous PA; * *p* < .05; ** *p* < .01; *** *p* < .001

### Results of the mediation models

In order to test hypothesis 4, three mediation models were tested in which the dependent variables were moderate PA, vigorous PA, and MVPA respectively. The results of the mediation analysis for **moderate PA** revealed that CR was associated with affective response to moderate PA (b = 0.73; *p* < .001), and that affective response to moderate PA was associated with moderate PA (b = 17.10; *p* < .05). The indirect effect was significant (b = 12.47; SE = 6.33; 95% CI: 2.16, 26.96) whereas the direct effect, the relationship between CR and moderate PA was no longer significant (b = 13.82; *p* = .26).

With regard to **vigorous PA**, CR was significantly associated with affective response to vigorous PA (b = 0.57; *p* < .05), and affective response to vigorous PA with vigorous PA (b = 15.81; *p* < .01). The indirect effect was significant (b = 9.04; SE = 5.81; 95% CI: 0.30, 55.28). CR was also significantly associated with vigorous PA (direct effect; b = 28.00; *p* < .05) while controlling for affective response.

Thirdly, with **MVPA** as dependent variable and inserting the two mediators (AR to moderate as well as vigorous PA) in parallel to the model, the results showed that CR was significantly associated with affective response to moderate (b = 0.73; *p* < .001) as well as vigorous PA (b = 0.57; *p* < .05). CR was associated with MVPA (direct effect; b = 50.20; *p* < .01). AR to vigorous (b = 23.70; *p* < .01) PA was associated with MVPA but not moderate PA (b = -0.57; *p* = .96). Overall, the indirect effect of CR on MVPA through the affective response to vigorous PA was significant (b = 13.54; SE = 8.67; 95% CI: 0.61, 34.33), while the indirect effect for affective response to moderate PA was not (b = -0.41; SE = 8.79; 95% CI: -17.53, 17.58).

## Discussion

The current study aimed to explore the role of CR and affective response to PA on PA behavior. Mainly, the correlational relationship among study variables were tested, and it was examined whether affective response mediates the relationship between CR and PA. Documented correlations were in the expected direction; CR was positively associated with affective response to both moderate and vigorous PA, affective response to moderate PA and to vigorous PA were positively correlated with the corresponding PA, and lastly CR was positively correlated with PA. Mediation analyses further revealed that affective response to moderate PA mediated the relationship between CR and moderate PA as affective response to vigorous PA mediated the relationship between CR and vigorous PA. Simply, CR predicted PA through affective response as expected. The more frequently CR was used, the more positive was the affective response to PA, and the more corresponding PA was engaged. These findings were in line with the current literature pointing that pleasantness of the affective response to PA is positively associated with PA [5–8]. Moreover, emotion regulation also plays an important role in regular PA. Changing the cognitive label that people ascribe to PA-related unpleasantness might help to reduce this unpleasantness, which might enhance the probability of repeating the behaviour. It could be argued that potentially there is a bidirectional relationship between emotion regulation and PA. With the use of CR, as the affective response becomes less negative, it contributes to PA engagement. Also, it has been supported that habitual exercise might enhance emotion regulation, specifically CR [16, 17] along with enhancing mood and cognition.

The results of the present study also showed that when the two mediators (affective response to moderate PA and vigorous PA) examined at once in their association with CR and MVPA, the indirect effect and thus mediation was significant for affective response to vigorous PA but not for moderate PA. In line with the earlier studies reporting that for intensity levels lower than vigorous PA produces improvements in positive affect [33], it can be argued that especially affective response to more intense PA, with the employment of CR strategies are more critical in the prediction of PA behavior. As higher intensity PA is typically associated with relatively less pleasant affective responses [15, 34], CR use could be mostly needed at higher intensity levels of PA. However, our results are at the same time in contrast to assumptions of the dual-mode theory of Ekkekakis [35] and empirical studies showing that cognitive reappraisal might become less effective as PA intensity approaches near-maximal efforts, as higher PA intensities (compared to lower intensities) are associated with lower neural activity in the prefrontal cortex, which is related to neural processes associated with cognitive reappraisal processes [36, 37].

### Implications

The results of the study suggest that enhancing the positive affective response to PA using CR strategies might be a beneficial tool in order to increase adherence to PA especially, more intense forms of PA. Studies in other domains showed that with CR interventions, CR ability and the frequency of CR use can be increased [38, 39]. Hence, in the PA domain, CR interventions can be implemented in PA promotion programs, especially for those who are currently inactive. Future research should aim to test the effectiveness of CR interventions in PA domain.

Future longitudinal and experimental research examining moment-by-moment changes in participants’ affect before, during, and after PA and CR engagement is needed to explore and better document the relationship between PA and CR.

### Limitations

We acknowledge several significant limitations in our study. Firstly, the use of a cross-sectional design restricts our ability to establish causal relationships between CR, affective response, PA behavior. Secondly, the assessment method of study variables poses another limitation, considering self-report subjective ratings. Specifically, the measurement of affect based on typical experiences, rather than during or after bouts of moderate or vigorous PA, may introduce recall bias and limit the accuracy of our findings. Thirdly, conducting mediation analysis with a small sample of cross-sectional data and relying on retrospective self-reported variables pose additional challenges. Lastly, the highly educated and predominantly women sample of the study diminishes the generalizability of the results.

## Conclusion

Despite limitations, the current study provided evidence in support of the prediction of PA behavior by CR through affective response. It is encouraged for future research to explore these relationships using longitudinal designs and objective measures of affect and physical activity.

## Data Availability

Data and materials will be shared upon reasonable request.

## Abbreviations

CR: Cognitive reappraisal
MVPA: Moderate-to-vigorous physical activity
PA: Physical activity
